# Complete Genome Sequence of *Acinetobacter baumannii* Strain B8300, Which Displays High Twitching Motility

**DOI:** 10.1128/genomeA.00956-15

**Published:** 2015-08-20

**Authors:** Saranya Vijaykumar, Veeraraghavan Balaji, Indranil Biswas

**Affiliations:** a Department of Clinical Microbiology, Christian Medical College, Vellore, Tamil Nadu, India; b Department of Microbiology, Molecular Genetics and Immunology, University of Kansas Medical Center, Kansas City, Kansas, USA

## Abstract

*Acinetobacter baumannii* has emerged as an important nosocomial pathogen causing health care-associated infections. In this study, we determined the genome of a twitching-positive clinical strain, B8300, isolated from a hospital in southern India. *De novo* assembly of PacBio long-read sequencing data generated the B8300 genome that consists of a chromosome of 3.82 Mbp and a plasmid of 25.15 kbp.

## GENOME ANNOUNCEMENT

*Acinetobacter baumannii*, a Gram-negative bacterium, has recently emerged as a nosocomial pathogen that causes a variety of infections, including bacteremia, meningitis, skin and soft tissue infections, pneumonia, and urinary tract infections (1). *A. baumannii* is often intrinsically resistant to many antibiotics and has the ability to uptake new resistance genes from the environment, making the infections difficult to treat (2). The organism is classically described as nonmotile; however, many clinical isolates display both swarming and twitching motilities (3).

We have isolated an *A. baumannii* strain (B8300) from the bloodstream infection of a 29-year-old male patient from Christian Medical College, Vellore, India. This isolate produces biofilm and displays extensive twitching motility but no swarming-like motility. The isolate is susceptible to all the commonly used antibiotics.

To facilitate molecular studies, we determined the complete genome sequence of *A. baumannii* B8300 using PacBio single-molecule real-time (SMRT) technology (4). We prepared a 3- to 20-kb genomic DNA library suitable for P6/C4 chemistry. Using one SMRT cell on the PacBio RSII sequencing platform, we obtained 79,883 reads with a mean read length of 10,446 bp. The reads were assembled *de novo* with Hierarchical Genome Assembly Process 3 (HGAP3) (5) within the SMRT Analysis version 2.3.0 software. The best assembly was selected, and Minimus 2 was used for trimming the circular contig (6). SMRT Analysis with the default parameters was used for detecting base modifications in the genome. The assembled B8300 genome consists of a single circular chromosome with 3,824,908 bp, containing 39.3% GC content, and a plasmid of 25,150 bp with 35.3% GC content.

The genome was annotated using BASys (7) and Analysis Engine at the University of Maryland (8). The genome predicts 3,711 open reading frames, 18 rRNA genes, 74 tRNA genes, and one transfer-messenger RNA (tmRNA). Three motifs with m6-adenosine and one motif with m4-cytosine methylations were detected in the genome. Sequence typing analysis at the CGE server (http://www.cbs.dtu.dk/services) indicated that B8300 did not match any known multilocus sequence types. ResFinder version 2.1 analysis at the CGE server returned only the *bla*_OXA-65_ gene and no other resistance genes in the B8300 genome. IslandViewer3 (9) analysis suggests that the B8300 genome harbors between 10 and 24 genomic islands. ISfinder analysis (10) indicates that the genome contains several IS elements, with the majority belonging to the IS*5* (IS*903* group) family. Similarly, PHAST analysis predicts four incomplete and one complete prophage sequences in the genome (11). CRISPRFinder analysis (12) suggests that the B8300 genome encodes two confirmed and possibly one additional clustered regularly interspaced short palindromic repeat (CRISPR) sequences. Finally, analysis by antiSMASH suggests the presence of four putative secondary metabolite gene clusters, including the arylpolyne, acinetobactin, and acinetoferrin biosynthetic gene clusters (13). The complete genome sequence of B8300 will be valuable for comparative genomic studies, understanding molecular mechanisms of motility, and developing molecular diagnostic tools to detect the pathogen.

### Nucleotide sequence accession numbers.

The *A. baumannii* B8300 genome sequence was deposited in GenBank under the accession number LFYY00000000. The version described in this manuscript is the first version, LFY00000000.1.
